# Zinc and selenium supplementation on treated HIV-infected individuals induces changes in body composition and on the expression of genes responsible of naïve CD8+ T cells function

**DOI:** 10.3389/fnut.2024.1417975

**Published:** 2024-09-16

**Authors:** Iván Armando Osuna-Padilla, Nadia Carolina Rodríguez-Moguel, Adriana Aguilar-Vargas, Maricruz Tolentino-Dolores, Otilia Perichart-Perera, Victor Ahumada-Topete, Santiago Ávila-Ríos, Maribel Soto-Nava, Dafné Diaz-Rivera, Enrique De León-Lara, Marti Wilson-Verdugo, Olivia Briceño

**Affiliations:** ^1^Coordinación de Nutrición Clínica, Departamento de Áreas Críticas, Instituto Nacional de Enfermedades Respiratorias “Ismael Cosío Villegas”, Mexico City, Mexico; ^2^Centro de Investigación en Enfermedades Infecciosas, Instituto Nacional de Enfermedades Respiratorias “Ismael Cosío Villegas”, Mexico City, Mexico; ^3^Coordinación de Nutrición y Bioprogramación, Instituto Nacional de Perinatología “Isidro Espinosa de los Reyes”, Mexico City, Mexico; ^4^Departamento de Biología Celular y del Desarrollo, Instituto de Fisiología Celular, Universidad Nacional Autónoma de México, Mexico City, Mexico

**Keywords:** micronutrient supplementation, zinc, selenium, HIV, antiretroviral therapy, body composition, immune system function

## Abstract

**Background and aim:**

Deficiency of zinc and selenium is common in persons living with human immunodeficiency virus (PLWHIV) and has been associated with the development of non-AIDS related comorbidities, impaired immune system function and mortality. Micronutrient supplementation on long-term-treated PLWHIV could bring potential clinical and immunological benefits improving their health status and quality of life. The aim of the present study is to analyze the effect of zinc and selenium supplementation on body composition, bone mineral density, CD4+ T-cell counts, metabolic profile and immune system status on clinical stable PLWHIV on long-term antiretroviral therapy (ART).

**Methods:**

This is a randomized pilot clinical trial in which we recruited 60 PLWHIV on ART who were assigned to the intervention groups: zinc (30 mg of zinc gluconate), selenium (200 μg of selenium yeast), zinc + selenium (same doses and presentations) or to a control group (without nutritional supplementation) who received supplementation during 6 months. Primary outcome was defined as changes in body composition (weight, muscle and fat mass and bone mineral density) and secondary outcomes as changes in biochemical and immunological parameters (CD4+ T-cell count, cholesterol, glucose, triglycerides and seric zinc and selenium seric concentrations) before and after supplementation. Peripheral blood mononuclear cells (PBMCs) of one individual of each intervention group were analyzed for single cell transcriptomics before and after supplementation.

**Results:**

BMI (*p* = 0.03), fat mass (*p* = 0.03), and trunk fat (*p* = 0.01) decreased after 6 months of selenium supplementation. No changes were observed for cholesterol, glucose or triglycerides after supplementation (*p* > 0.05 in all cases). CD4+ T cells percentage increased after 6 months of selenium supplementation (*p* = 0.03). On the transcriptome analysis, zinc and selenium supplementation induced changes on de expression of genes associated with the function of naive and memory CD8+ T-cells (*p* < 0.05 in all cases).

**Conclusion:**

Zinc and selenium supplementation could represent a complementary intervention that may improve the health status and immune response of treated PLWHIV.

## Introduction

Micronutrient deficiencies are common among PLWHIV due to suboptimal dietary intake and increased requirements or losses driven by chronic diarrhea, malabsorption and chronic inflammation (1). Micronutrient deficiencies have been associated with inappropriate immune system function, HIV disease progression, mortality and also with the development of non-AIDS related comorbidities on treated PLWHIV (2).

Particularly, low zinc and selenium seric concentrations have been reported on PLWHIV, even among those who are on long-term ART on virological control (3). Zinc deficiency has been associated with multiple parameters of HIV disease progression, such as systemic inflammation, increased intracellular viral replication and decreased CD4+ T cell counts (4). On the other hand, selenium deficiency has been associated with HIV disease progression and mortality (5), moreover, its deficiency in non-HIV-infected population has been implicated in the development of metabolic diseases including cardiovascular disease, type 2-diabetes mellitus and insulin resistance (6).

Some clinical trials have studied the effect of zinc and selenium supplementation on PLWHIV with controversial results, due to the diversity on the clinical characteristic of the cohort, dosage and presentation of the supplements, duration of supplementation and the clinical outcomes of the studies. It has been shown that zinc gluconate containing 30 mg of elemental zinc, has the highest bioavailability compared to other zinc compounds (7) and that 200 μg of selenium yeast per day showed to rise the plasma selenium concentration efficiently (8). Additionally the safety of zinc or selenium supplementation when administrated during 6 months in PLWHIV (9) has been demonstrated, while previous reports have observed poor effect of supplementation during shorter periods of time (10, 11).

On a metanalysis, zinc supplementation on PLWHIV has shown to delay disease progression and decrease the rate of opportunistic infections in individuals with or without ART (12). There are controversial reports about the effect of zinc supplementation on CD4+ T cell replenishment. Some authors reported no effect of zinc supplementation on CD4+ T cells levels (13–15). In contrast, Asdamongkol et al. and others, found increased CD4+ T cell counts after 6 months of zinc supplementation (16, 17). Moreover, some studies have reported therapeutic benefits of zinc supplementation in non-HIV-infected population, this via reduction of inflammation and oxidative stress (18).

Selenium supplementation in PLWHIV has been associated with decrease in CD4+ T cell decline, delay progression to AIDS and lower diarrheal morbidity (9). Some authors have reported increased CD4+ T cell numbers after 18 months of selenium supplementation (19), while others did not find any effect (20). Additionally, selenium has shown to reduce the immune activation and inflammation (21, 22), which have been associated with HIV disease progression, development of non-AIDS related comorbidities and mortality (23).

The implementation of intervention strategies, such as nutritional counselling and providing zinc and selenium supplementation to complement the diets of clinically stable PLWHIV on long term ART, could have potential benefits on improving the immune system function and impeding or delaying the onset of non-AIDS associated comorbidities, resulting in improvement of their health status and overall quality of life. Thus, the aim of this study was to assess the effect of zinc and selenium supplementation on body composition, bone mineral density, CD4+ T cell replenishment, lipid profile, glucose levels and immune system status in PLWHIV on ART without metabolic diseases.

## Materials and methods

### Study design and participants

This four arms randomized pilot clinical trial was conducted from September 2016 to December 2019 on patients with documented HIV infection (as per National Guidelines using two fourth-generation ELISA (24)) under virologic control. All patients were enrolled at the National Institute of Respiratory Diseases, Mexico City. Eligible subjects were all males, ≥18 years old, CD4+ T cells >200 cell/mm^3^, on ART for at least 2 years and with undetectable viral load (<40 HIV RNA copies/ml). Individuals were invited to participate if they had not been on a diet to lose or gain weight 3 months prior to the study. Exclusion criteria were: malnutrition or non-communicable disease diagnosis at the moment of recruitment, taking oral hypoglycemic agents, antihypertensive drugs, or lipid-lowering treatment or consumption of any nutritional or antioxidant supplements 3 months prior to the study. Elimination criteria were: low adherence to ART or to supplementation or presenting active opportunistic infections or intolerance to supplementation manifested by nausea, vomiting, diarrhea or dermatitis at any time during follow-up. We did not collect additional information about other self-reported adverse events and we did not observe any deaths or serious adverse events during the entire time of the intervention. The Ethics and Biosecurity Committees of the National Institute of Respiratory Diseases approved the protocol (C21-16). This study was registered in ClinicalTrials.gov (NCT03421314). The study was conducted according to the principles expressed in the Declaration of Helsinki. All participants signed a written informed consent at the moment of recruitment.

### Intervention

Supplementation was administered during 6 months based on previous reports showing the safety of zinc and selenium supplementation in PLWHIV during this period of time (9). Supplementation dosage was prescribed according to ESPEN guidelines recommendations (25). Individuals in the zinc group received a daily dose of zinc gluconate (Nutra Manufacturing INC, USA) containing 30 mg of elemental zinc, as it has been demonstrated that this presentation has the highest bioavailability compared to other zinc compounds (7). Individuals in the selenium group received 200 μg of selenium yeast per day (2 tablets with 100 μg of selenium each; Nutra Manufacturing INC, USA), as this dose and chemical form of selenium showed to rise the plasma selenium concentration efficiently (8). Individuals in the zinc + selenium group received 30 mg of elemental zinc + 200 μg of selenium per day of the same presentation. All supplements were provided in standard sealed plastic containers. Patients in the control group were observed for the same period of time without any nutritional supplementation or administration of placebo tablets. We gave 35 tablets to the individuals into the zinc intervention group (1 per day + 5 extra tablets in case of reschedule the follow-up), 70 tablets to the individuals into the selenium intervention group (2 per day + 10 extra tablets) and 105 tablets to the individuals into the zinc + selenium intervention group (1 zinc pill per day + 5 extra tablets, and 2 selenium pills per day + 10 extra tablets) and were restocked each month by a dietitian who was not involved into the clinical follow-up of the participants. Good Adherence to supplementation was assessed monthly and was considered acceptable if less than 10% of the total number of tablets were returned by the patients. In addition, monthly nutritional counseling (focused on healthy eating habits) and clinical follow-up with an infectious disease specialist were performed to identify comorbidities, opportunistic infections or adverse effects related to supplementation such as nausea, vomiting, diarrhea or dermatitis. The nutritional counselling and clinical follow-up by an infectious disease specialist are routinary performed each 6 months in our hospital as part of the standard clinical follow-up of PLWHIV.

### Outcomes

Primary outcome was defined as changes in body composition (weight, muscle and fat mass, and bone mineral density) and secondary outcomes were changes in biochemical and immunological parameters (CD4+ T-cell count, cholesterol, glucose, triglycerides, seric zinc and selenium concentrations and transcriptome of immune cells) before and after supplementation.

### Body composition and bone mineral density assessment (BMD)

Body weight and height were measured using a digital scale with a precision of 0.1 kg (SECA 769, Munich, Germany) and a wall-mounted stadiometer graduated in centimeters with a precision of 0.1 cm (SECA 206, Munich, Germany), respectively. Both measurements were used for calculating body mass index (BMI). Body composition was assessed by a certified technician using dual-energy X-ray absorptiometry (DXA; Lunar Prodigy Advance, GE Health Care, Little Chalfont, UK) with the patient in a supine position. System quality assurance protocols were performed daily by the manufacturer’s instructions. Muscle mass, total-fat mass in kilograms and percentage were calculated using enCORE 2010 software using scan modes (thick, standard, or thin) suggested by the software. At the end of the scan, all body composition analyses were thoroughly checked for random measurement errors (i.e., regions of interest errors or metal artifacts) and were manually adjusted for the region of interest for regional estimations (arms, legs, and trunk). Bone mineral density (BMD) was measured in lumbar and femoral regions and was reported as g/cm^2^. The measurements of body composition and BMD are routinary performed in our hospital at least each 6 months and annually, respectively, as part of the standard clinical follow-up of PLWHIV.

### Dietary assessment

Dietary intake was assessed before and after supplementation using a 3-day food record (one weekend day and two weekdays). Participants were instructed to report all foods and beverages consumed. Data was corroborated through face-to-face interviews by a trained dietitian, and portion sizes were quantified using household measures (spoons, plates, cups, and glasses) and food models. The data collected were: amount of foods consumed, brand of processed foods, use of condiments or added fat, recipes, and culinary methods. Conversion of food intake into nutrients was made using the computer software Food Processor Plus (Esha Research, Salem, OR, USA). The nutritional content of typical Mexican food was added to the original database. Food records were entered into the software and average energy (kcal), zinc (mg), and selenium (μg) daily intake were determined. Low daily intake of zinc and selenium were defined according to the estimated average requirement (EAR) established by the Institute of Medicine of the National Academies; EAR values were <9.4 mg/day for zinc and <45 μg/day for selenium. Dietary assessment and nutritional counseling are routinary performed in our hospital at least every 6 months, as part of the standard clinical follow-up of PLWHIV.

### Biochemical determinations

24 ml of fasting blood were drawn to measure total cholesterol, HDL-Cholesterol, LDL-Cholesterol, glucose, and triglycerides using standard automated techniques at the diagnostic laboratory of the National Institute of Respiratory Diseases. Metabolic disorders were defined as total cholesterol >200 mg/dl, glucose >100 mg/dl, triglycerides >150 mg/dl, HDL-Cholesterol <40 mg/dl and LDL-Cholesterol>110 mg/dl as previously reported (26). Biochemical measurements are performed at least twice per year or by request of infectious disease specialist. This assessment is considered as part of the standard clinical follow-up of PLWHIV in our hospital.

### Plasma viral load and CD4+ T cell counts

6 ml of fasting blood were drawn to determine plasma HIV viral load by automated real-time polymerase chain reaction (PCR) using the m2000 system (Abbott, Abbott Park, IL, USA) with detection limit of 40 HIV-1 RNA copies/ml. CD4+ T-cell counts were determined by flow cytometry using the Trucount Kit in FACSCanto II instruments (BD Biosciences, San Jose, CA, USA). Plasma viral load and CD4+ T cells counts are assessed in our hospital as part of the standard clinical follow-up of PLWHIV annually in accordance to an infectious disease specialist indication. Only for research proposes, they were performed each 6 months for the participants of this study.

### Zinc and selenium seric determinations

6 ml of fasting blood were drawn to quantified seric zinc using the Perkin-Elmer Analyst 400 atomic absorption spectrometer (Norwalk, CT, USA) by the method of standard additions. Seric selenium concentration was measured by Perkin-Elmer 2,100 DV optical emission inductively couple plasma spectrometer by hydride generation. Zinc deficiency was defined as a level < 75 mg/dl and selenium deficiency as a level < 85 μg/L, based on a previous study (3). Zinc and selenium seric determinations are not routinary performed in our hospital as part of the standard clinical follow-up of PLWHIV and were only considered for research proposes for the participants of this study.

### CD4+ and CD8+ T cells immune activation

Peripheral blood mononuclear cells (PBMCs) were isolated from 12 ml of peripheral blood by density gradient centrifugation using lymphoprep (Axis-Shield, Oslo, Norway) according to manufacturer’s instructions. Vials with 25 million PBMCs were cryopreserved until used. After thawing, total PBMCs were manually counted and washed with phosphate-buffered saline (PBS, Lonza, CA, USA). A total of 5 million PBMCs were stained extracellularly by incubating the cells with an antibody cocktail at room temperature for 15 min. Finally, cells were fixed in 300ul of 1% paraformaldehyde (Sigma-Aldrich, St. Louis, MO, USA) and acquired immediately on a FACS LSR II cytometer (BD Biosciences, CA, USA). The following immunofluorescent monoclonal antibodies were used: BV570-CD3 (clone UCHT1), APC-Cy7-CD4 (clone A161A1), BV711-CD38 (clone HIT2), BV785-HLADR (clone L243) from Biolegend (San Diego, CA, USA) and Alexa Fluor 700-CD8 (clone RPA-T8) from BD Pharmingen (Franklin Lakes, NJ, USA). Live/dead aqua fluorescent reactive dye, from Invitrogen (Thermo Fisher scientific, Waltham, MA, USA) was used as a viability marker. Fluorescence minus one (FMO) stained tubes were used as gating controls. The software FlowJo 10 was used to analyze the data following the next strategy of gating: after using cleaning gates for singlets, morphology and viability, immune activation status (CD38/HLADR co-expression) was analyzed on total CD3+ CD4+ and CD3+ CD8+ T cells. CD4+ and CD8+ T cells immune activation determination is not routinary performed in our hospital as part of the standard clinical follow-up of PLWHIV and were only performed for research proposes for the participants of this study.

### Single cell RNA-seq library preparation

We used frozen PBMCs samples from baseline (t = 0) and at 6 months after receiving supplementation (t = 6) from the zinc, selenium or control groups (1 individual of each group that experienced a significant increase on the seric concentration of zinc and selenium after 6 months of supplementation was selected). The group supplemented with both zinc+selenium was not included in this experiment. The PBMCs were thawed and incubated overnight at 37°C with RPMI (PBS, Lonza, CA, USA) with 10% bovine serum (Hyclone Merck, Darmstadt, Germany). The next day, the samples were labeled with molecular markers using Sample Tag (1–7) BD Human Single Cell Multiplexing Kit (Cat, 633,781, San Jose, California, USA) following manufacturer’s instructions. Then, the samples were sorted to purify live/CD45(+)/CD15(−) cells (using antibodies from Biolegend; San Diego, CA, USA). These cells were pulled together to ensure analysis of at least 5,700 cells from each sample with a total of 40,000 cells. To capture mRNA, we used the BD Rhapsody Express Single Cell Analysis System, following manufacturer’s instructions for the BD Rhapsody Express Targeted library workflow. Briefly, the sorted cells were loaded on the cartridge with the Cell Capture Beads provided by the kit; then, the cells were lysed to capture all messenger RNA. Finally, cDNA synthesis and the library preparation were performed following manufacturer’s instructions from the BD Rhapsody Immune Response Targeted Panel (Human) kit, (Cat, 633,750, San Jose, California, USA), which contains 399 genes for profiling human immune cells: naïve and memory CD4+ T cells, naïve and memory CD8+ T cells, Monocytes, Dendritic Cells, gamma-delta T cells, mast cells, B cells, NK and NKT cells.

### Single cell RNA-seq data analysis

The FASTQ files obtained from sequencing were processed following the BD Rhapsody Targeted Analysis Pipeline (BD Biosciences). First, low quality read pairs were removed based on read length, base calling quality and highest single nucleotide frequency. Filtered R1 reads were used to identify Unique Molecular Identifier (UMI) sequences. Then, read pairs were aligned to the Rhapsody Immune Response Panel Hs (BD Biosciences) reference using STAR (27).The generated raw count matrices were adjusted by two error correction algorithms developed by BD Biosciences—recursive substitution error correction (RSEC) and distribution-based error correction (DBEC). Unique cells were then estimated using the second derivative analysis to filter out noise cell labels, based on the assumption that putative cells have many more reads than noise cell labels. Thus, the cells were sorted in the descending order by the number of reads and the inflexion point can be observed on a log-transformed cumulative curve of the number of reads. Cell type labels were obtained for each cell using a machine learning model developed on human PBMCs (BD Biosciences), and cells with an undetermined type label were excluded from further analyses. The DBEC-adjusted read count matrices obtained from the Rhapsody pipeline were further analyzed using Seurat (28). Expression matrices were log normalized and dimensional reduction was performed using the first 30 principal components. Uniform Manifold Approximation and Projection (UMAP) was used for data visualization. Data was scaled using the Seurat Scale Data function for integrative analysis between samples. Differential gene expression analysis between conditions was performed using the Seurat Find Markers function, using an adjusted *p*-value threshold of less than 0.05 for a gene to be considered differentially expressed. For the longitudinal analysis of selenium and zinc supplementations, we considered a gene to be differentially expressed if it fulfilled all of the following conditions: (a) it was differentially expressed in the supplementation (either zinc or selenium) t0 vs. supplementation t6 comparison, (b) it was differentially expressed in the control t6 vs. supplementation t6 comparison, (c) it was not differentially expressed in the control t0 vs. control t6 comparison, (d) it was not differentially expressed in the control t0 vs. supplementation t0 comparison. Heatmaps of differentially expressed genes were generated with the R package Pheatmap, using the average expression values of genes by sample and cell type, which were obtained using the Seurat Average Expression function.

### Sample size

For the calculation of sample size, we obtained data from a published article in which a 13% of variation of selenium levels was observed after their supplementation. We used a statistical power of the 80% with a significance <0.05 in the formula using R studio software and we got a sample size of 84 individuals. Due to the COVID-19 pandemic, recruitment and follow-up could not be completed; therefore, we modified the original design from a clinical trial into a pilot clinical trial. The final number of individuals per group in the present study was: G1 (zinc supplementation) *n* = 18, G2 (selenium supplementation) *n* = 13, G3 (zinc + selenium supplementation) *n* = 13 and G4 (control group) *n* = 16.

### Randomization and blinding

Potential participants were primarily identified by health providers who invited them to join the study. Participants were randomly assigned to either zinc group, selenium group, zinc + selenium group or control group using a dynamic randomization method designed to be balanced by the following strata: age, CD4+ T cell count, CD4+ T cells nadir and time on ART. R Studio software was used for this purpose using covariate-adaptative randomization with the carat package. Patients who were randomized into supplementation groups and clinic staff were blinded during the complete follow-up.

### Statistical analysis

Statistical analyses were performed using STATA software version 14 (Stata Corp, College Station, TX, USA). Data distribution of continuous variables was examined with the Saphiro Wilk test. Values are expressed as mean ± SD for normally distributed continuous variables and median (interquartile ranges [IQRs]) for skewed variables or percentage of total for categorical variables. Two-way analysis of variance for repeated measures with Bonferroni’s multiple comparisons test was used to compare body composition, clinical, biochemical and immunological variables between supplementation groups. Furthermore, within group differences were assessed using the paired *t*-test (or Wilcoxon signed ranks test). Linear (β, CI95%) regression models were performed to assess the association between supplementation group and fat mass loss (in % and kilograms) adjusted to baseline value of body composition. *p*-values less than 0.05 were considered significant.

## Results

### Cohort characteristics before supplementation

We recruited 60 men diagnosed with HIV, on long-term ART, who were randomly assigned into three supplementation groups (G1, assigned to zinc supplementation, *n* = 18; G2, assigned to selenium supplementation, *n* = 13; G3, assigned to zinc + selenium supplementation, *n* = 13) and a control group without supplementation (G4, *n* = 16). The median of age in the entire cohort was 39.5 ± 9.4 years and we did not find statistical age differences between the study groups (*p* > 0.05 in all cases). Additionally, the mean time since HIV diagnosis (6 years), mean time receiving ART (4.8 years) and the mean nadir CD4+ T cells counts (161 cells/mm^3^) were similar between the 4 study groups (*p* > 0.05 in all cases). The mean CD4+ T cell count of the entire cohort was 580 ± 234 cells/mm^3^ without statistical difference between the study groups (*p* > 0.05 in all cases). Also, we did no find differences in the relative abundance of CD4+ T cells between groups (*p* > 0.05 in all cases). All the individuals recruited had undetectable viral load (<40 HIV RNA copies/ml). Forty-four participants (77%) from the entire cohort were on Efavirenz (EFV) + Emtricitabine (FTC) + Tenofovir disoproxil fumarate (TDF); only 4 (6%) were on Abacavir/Lamivudine (ABC/3TC) + Efavirenz (EFV) and 12 (17%) were on different regimens. We did not find differences in the ART regimen when the study groups were compared (*p* > 0.05 in all cases; Table 1). We found that the 4 studied groups had similar amounts of energy, zinc and selenium intake and also the seric levels of zinc and selenium were similar in the study groups before supplementation (*p* > 0.05 in all cases; Table 1). Moreover, we observed that 48.5% of the entire cohort presented suboptimal zinc intake (<9.4 mg/day), while only 4% of the cohort showed suboptimal selenium intake (<45 μg/day). When the seric levels of zinc and selenium were analyzed, we found that 19 and 46.5% of the entire cohort showed zinc and selenium deficiency respectively; however, the individuals that presented suboptimal intake or seric deficiency were homogeneously distributed in the 4 groups of study (*p* > 0.05 in all cases; Table 1).

**Table 1:** Cohort characteristics of PLWHIV before supplementation.

Clinic characteristics					
	G1 assigned to zinc supplementation (*n* = 18)	G2 assigned to selenium supplementation (*n* = 13)	G3 assigned to zinc + selenium supplementation (*n* = 13)	G4 assigned to control (*n* = 16)	*p* value
Age (years)	40 ± 11.2	40.9 ± 9.2	37.8 ± 8.2	39.3 ± 8.8	0.86^a^
Time since HIV diagnosis (years)	8 (5–15)	6 (3–10)	4 (3–9)	6 (5–9)	0.18^b^
Time on ART (years)	4.5 (3–12)	5 (3–9)	4 (3–8)	6 (5–8.5)	0.64^b^
Nadir CD4+ T cell count (cells/mm^3^)	289 (123–452)	169 (16–319)	86 (52–135)	100 (17–299)	0.50^b^
CD4+ T cells count (cells/mm^3^)	478 (360–713)	572 (358–627)	468 (404–619)	654 (477–832)	0.51^b^
CD4+ T cells (%)	28 ± 8	29 ± 12	30 ± 10	30 ± 8	0.96^a^
Viral Load (HIV-1 RNA copies/ml)	<40	<40	<40	<40	
ART Regimen EFV + FTC + TDF ABC/3TC + EFV Others	67% 0% 33%	69% 8% 23%	61% 15% 24%	94% 0% 6%	0.28^c^
Dietary measurements					
Energy intake (kcal/day)	2,263 ± 686	2,517 ± 658	2,245 ± 468	2,541 ± 575	0.54^a^
Dietary Zinc intake (mg/day) Suboptimal intake (<9.4 mg)	8.9 (6.7–10.9) 10 (56%)	12.2 (6.8–16) 6 (46%)	8.7 (7.4–12.9) 7 (54%)	10.6 (9.3–12.5) 6 (38%)	0.41^b^ 0.72^c^
Dietary Selenium intake (μg/day) Suboptimal intake (<45 μg)	90.9 (62.3–119.3) 2 (11%)	119 (89–133.1) 1 (8%)	122 (64.4–152.2) 0%	104.9 (87–146.2) 0%	0.44^b^ 0.37^c^
Seric Zinc (mg/dl) Deficiency % (<75 mg/dl)	85 ± 14.4 4 (22%)	85.3 ± 12.1 2 (15%)	89.7 ± 12.9 1 (8%)	82.1 ± 12.3 5 (31%)	0.48^a^ 0.43^c^
Seric Selenium (μg/dl) Deficiency % (<85 μg/dl)	86.5 ± 9.5 6 (35%)	84.9 ± 10.5 4 (40%)	83.7 ± 9.9 7 (58%)	83.9 ± 12.4 8 (53%)	0.87^a^ 0.57^c^

ART, antiretroviral treatment; EFV, efavirenz; FTC, Emtricitabine; TDF, Tenofovir disoproxil fumarate; ABC, Abacavir; 3TC, Lamivudine.

a One way ANOVA.

b Kruskal-Wallis test.

c *X2 test.*

We measured weight, body mass index (BMI), fat mass (absolute and relative), trunk fat, muscle mass and bone mineral density (BMD, femoral and lumbar) in order to characterize the body composition of the cohort at baseline before supplementation. Fat mass at baseline (expressed in either percentage or kilograms) was significantly higher in the G2 (assigned to selenium supplementation) compared to G3 (assigned to zinc + selenium supplementation; *p* = 0.01).

Additionally, the G1 (assigned to zinc supplementation) had significantly lower lumbar BMD than G2 (assigned to selenium supplementation; *p* = 0.03) before supplementation. We did not find differences on the weight, BMI, trunk fat mass, muscle mass or femoral BMD between the studied groups before supplementation (*p* > 0.05 in all cases; Supplementary Table 1). We also evaluated the blood biochemical parameters: total cholesterol, HDL, LDL, triglycerides and fasting glucose in order to determine de presence of metabolic diseases in the cohort at baseline before supplementation. We found that the levels of total cholesterol, HDL, LDL, triglycerides and glucose before supplementation were not different between the studied groups (*p* > 0.05 in all cases). Also, the prevalence of hypercholesterolemia, low HDL, high LDL, hypertriglicerydemia or impaired glucose levels was similar between the 4 studied groups before supplementation (*p* > 0.05 in all cases; Supplementary Table 1).

### Changes in body composition, clinical, biochemical and immunological outcomes after 6 months of zinc or/and selenium supplementation

All the individuals that were included in the present analysis completed the supplementation with more than 90% of adherence to the zinc and/or selenium treatment during the entire time of the intervention. Moreover, after 6 months of supplementation with zinc, selenium or both nutriments simultaneously, any of the participants developed side effects such as nauseas, vomiting, diarrhea or dermatitis associated to the intervention. Any of the participants of the study died or developed serious adverse events during the entire intervention time.

We found that the individuals that were supplemented with selenium significantly decreased the fat mass in kilograms (*p* = 0.03) and percentage (*p* = 0.02) as well as their trunk fat (*p* = 0.01) and BMI (*p* = 0.03; Table 2, panel A). These changes were assesses using linear regression analysis, and we found that selenium group significantly decreased fat mass in kilograms (β 1.7, CI95% 0.20–3.3, *p* = 0.02) adjusted to basal value in comparison to another supplementation group. Similar result was observed for fat mass in percentage (β 2.1, CI95% 0.34–3.9, *p* = 0.02). We did not observe any significant differences on body composition parameters or BMD after 6 months of supplementation with zinc or zinc + selenium (*p* > 0.05 in all cases; Table 2, panel A).

**Table 2:** Changes on body composition, bone mineral density and biochemical measurements after 6 months of zinc or/and selenium supplementation.

	G1 = Zinc supplemented group (*n* = 18)			G2 = Selenium supplemented group (*n* = 13)			G3 = Zinc + Selenium supplemented group (*n* = 13)			G4 = No supplemented Control group (*n* = 16)			ANOVA
	Baseline	Six-Months	*p*	Baseline	Six-Months	*p*	Baseline	Six-Months	*p*	Baseline	Six-Months	*p*	*p*
A. Body composition and bone mineral density measurements													
Weight (kg)	66.3 ± 7.9	66.4 ± 8.2	0.89	70.9 ± 10.9	69.2 ± 11.3	0.08	68.6 ± 7.7	68.3 ± 8.2	0.79	71.8 ± 12.0	71.9 ± 12.1	0.91	0.43
BMI (kg/m^2^)	23.0 ± 2.6	23.0 ± 2.7	0.87	**24.6 ± 4.1**	**23.8 ± 4.3**	**0.03**	23.7 ± 2.8	23.1 ± 2.3	0.29	24.4 ± 3.4	24.4 ± 3.6	0.93	0.16
Fat mass (%)	23.9 ± 3.8	23.8 ± 4.4	0.82	**28.6 ± 4.5**	**26.4 ± 5.9**	**0.02**	23.0 ± 5.3	22.5 ± 4.6	0.58	27.1 ± 5.8	27.4 ± 6.9	0.68	0.06
Fat mass (kg)	16.1 ± 3.8	15.9 ± 4.0	0.66	**20.6 ± 6.3**	**18.8 ± 6.5**	**0.03**	16.0 ± 5.0	15.6 ± 4.7	0.65	19.7 ± 6.1	20.1 ± 7.2	0.49	0.06
Trunk fat mass (kg)	9.1 ±2.8	9.7 ±3.1	0.25	**11.6 ± 4.0**	**10.4 ± 4.4**	**0.01**	8.8 ±3.4	8.5 ±3.1	0.66	11.3 ± 4.0	11.6 ± 4.5	0.36	0.05
Muscle mass (kg)	48.2 ± 5.8	48.3 ± 6.2	0.85	47.5 ± 5.4	48.0 ± 5.8	0.26	50.3 ± 5.4	50.3 ± 5.5	0.96	49.5 ± 7.7	49.3 ± 6.8	0.67	0.76
Femoral BMD (g/cm^2^)	0.93 ± 0.14	0.95 ± 0.03	0.80	1.02 ± 0.15	1.02 ± 0.15	0.74	0.96 ± 0.09	0.96 ± 0.08	0.84	0.99 ± 0.07	0.98 ± 0.07	0.37	0.38
Lumbar BMD (g/cm^2^)	1.04 ± 0.14	1.05 ± 0.15	0.34	1.17 ± 0.13	1.19 ± 0.13	0.19	1.10 ± 0.10	1.10 ± 0.09	0.67	1.12 ± 0.11	1.11 ± 0.11	0.75	0.42
B. Biochemical measurements													
Total cholesterol (mg/dl)	168.3 ± 33.8	172.5 ± 34.7	0.35	182.8 ± 39	175.3 ± 46.9	0.12	182.7 ± 27.2	189.8 ± 25.5	0.33	175.1 ± 42.8	178.3 ± 39.6	0.65	0.42
HDL cholesterol (mg/dl)	42.8 ± 8.7	44.5 ± 9.8	0.19	43.6 ± 7.0	45.8 ± 10.4	0.08	43.7 ± 11.2	43.6 ± 7.8	0.93	43.5 ± 9.1	44.3 ± 8.5	0.72	0.85
LDL-cholesterol (mg/dl)	100.3 ± 27.8	106.5 ± 24.4	0.09	114.4 ± 31.2	106.1 ± 35.6	0.09	114. ± 22.7	117.9 ± 15.7	0.61	103.9 ± 86.8	108.1 ± 91.4	0.41	0.31
Triglycerides (mg/dl)	138.5 (97–210)	155 (112–194)	0.98	138.2 (81–164.5)	104.8 (81.7–140)	0.10	155.5 (105–227.9)	133.6 (112–228.5)	0.43	125.1 (99.8–266.1)	143.9 (106.3–167.3)	0.99	0.56
Glucose (mg/dl)	88 (83–94)	89 (80–97)	0.84	85 (82–87)	88 (84–93)	0.90	88 (86–94)	89 (87–93)	0.36	89 (86–97)	89 (86–95)	0.42	0.97

Kruskal-Wallis or student *t* test was performed for comparison between groups. BMI, body mass index; BMD, Bone mineral density; ND, non determined; HDL, high density lipoprotein, LDL, low density lipoprotein. Statistically different values are highlighted in bold.

No changes on the total cholesterol, HDL or LDL levels, triglycerides or glucose after 6 months of zinc, selenium or simultaneously both micronutrients supplementation independently of serum zinc or selenium deficiency at baseline (*p* > 0.05 in all cases; Table 2, panel B). Additionally, we analyze the effect of zinc or/and selenium supplementation on these biochemical parameters, but only on the individuals with baseline seric zinc or selenium deficiency. We did not find any effect of zinc or/and selenium supplementation on total cholesterol, HDL or LDL levels, triglycerides or glucose in the individuals with baseline zinc (*p* > 0.05 in all cases; Supplementary Table 2) or selenium (*p* > 0.05 in all cases; Supplementary Table 3) deficiency.

The measurements of the dietary intake showed that the individuals in the 4 groups of study did not change the amount calories (energy) or intake of zinc or selenium in their diets during the 6 months of the intervention (*p* > 0.05 in all cases; Table 3, panel A). Nonetheless, as expected, the seric levels of zinc, selenium or both micronutrients increased after 6 months of supplementation, but without reaching statistical difference (*p* > 0.05 in all cases; Table 3, panel A). In all the cases, the final concentration of seric zinc or/and selenium reached normal ranges after supplementation.

**Table 3:** Changes on dietary and immunological measurements after 6 months of zinc or/and selenium supplementation.

	**G1** = Zinc supplemented group (*n* = 18)			G2 = Selenium supplemented group (*n* = 13)			G3 = Zinc + Selenium supplemented group (*n* = 13)			G4 = No supplemented control group (*n* = 16)			ANOVA
	Baseline	Six-Months	*p*	Baseline	Six-Months	*p*	Baseline	Six-Months	*p*	Baseline	Six-Months	*p*	*p*
A. Dietary measurements													
Energy intake (kcal/day)	2,263 ± 686	1962 ± 353	0.10	2,517 ± 658	2050 ± 496	0.06	2,245 ± 468	2,213 ± 593	0.88	2,541 ± 575	2,410 ± 387	0.46	0.34
Zinc intake (mg/day)	8.9 (6.7–10.6)	10.3 (7.8–11.3)	0.44	12.2 (6.8–16)	7.8 (5.7–9.1)	**0.26**	8.6 (7.4–12.9)	8.4 (6.5–11.3)	0.70	10.6 (9.2–12.5)	10.7 (7–12.5)	0.44	0.06
Selenium intake (μg/day)	90.9 (62.3–119.3)	97.6 (74.3–123.3)	0.94	119 (89.6–133.1)	103.4 (79.3–106.8)	0.60	122 (64.4–152.2)	106.8 (85.8–111.4)	0.91	104.9 (87–146.2)	114.8 (75.2–142.1)	0.88	0.74
Zinc intake + supplementation (mg/day)	8.9 (6.7–10.6)	40.3 (37.8–41.3)	<0.001	ND	ND		8.6 (7.4–12.9)	38.4 (36.5–41.3)	<0.001	10.6 (9.2–12.5)	10.7 (7–12.5)	0.44	<0.001
Selenium intake + supplementation (μg/day)	ND	ND		119 (89.6–133.1)	303.4 (279.3–306.8)	<0.001	122 (64.4–152.2)	306.8 (285.8–311.4)	<0.001	104.9 (87–146.2)	114.8 (75.2–142.1)	0.88	<0.001
Seric zinc (mg/dl)	86.5 ± 14.2	94.7 ± 21.9	0.15	ND	ND		89.4 ± 13.4	93.7 ± 16.2	0.41	83.0 ± 12.1	83.9 ± 13.3	0.85	0.65
Seric Selenium (μg/dl)	ND	ND		82.6 ± 10.6	91.1 ± 11.1	0.22	83.7 ± 9.9	88.7 ± 11.6	0.17	84 ± 12.8	85.4 ± 12.8	0.06	0.81
B. Immunological measurements													
CD4+ (cells/mm^3^)	478 (360–713)	545 (368–768)	0.05	553 (358–627)	469 (356–687)	0.87	507 (404–680)	475 (379–836)	0.13	655 (478–832)	713 (457–802)	0.55	0.79
CD4 (%)	28.3 ± 7.6	28.2 ± 7.7	0.87	**28.8 ± 11.7**	**31.1 ± 12.8**	**0.03**	29.7 ± 9.3	28.7 ± 6.6	0.33	29.8 ± 8.3	31.2 ± 9.2	0.06	0.86
CD4+ Tcells [CD38 + HLADR+ (%)]	4.7 ± 1.6	4.7 ± 1.9	0.98	5.3 ± 2.5	5.0 ± 2.4	0.66	5.4 ± 2.5	4.7 ± 1.4	0.32	5.3 ± 2.7	4.2 ± 3.1	0.22	0.70
CD8+ Tcells [CD38 + HLADR+ (%)]	5.3 ± 3.9	5.8 ± 4.6	0.54	4.5 ± 4.7	3.8 ± 3.5	0.29	7.1 ± 5.3	6.2 ± 5.7	0.46	5.7 ± 4.3	4.8 ± 5.0	0.51	0.70

Kruskal-Wallis or student *t* test was performed for comparison between groups. BMI, body mass index; BMD, bone mineral density; ND, non determined; HDL, high density lipoprotein; LDL, low density lipoprotein.

Meanwhile, in the analyses of the immunological data, we found that CD4+ T cell counts tend to increase after zinc supplementation (*p* = 0.05) while CD4+ T cells frequency significantly increased after selenium supplementation (*p* = 0.03; Table 3, panel B). When we analyzed the effect of zinc or/and selenium supplementation on CD4+ T cell counts only in the individuals with baseline seric zinc or selenium deficiency, we found no significant changes on CD4+ T cells counts or frequency after supplementation (*p* > 0.05 in all cases; Supplementary Tables 2, 3). The frequency of activated (CD38 + HLADR+) CD4+ and CD8+ T cells did not change after 6 months of supplementation with zinc, selenium or both micronutrients simultaneously (*p* > 0.05 in all cases; Table 3, panel B).

### Single cell transcriptome analysis

**Figure 1:**
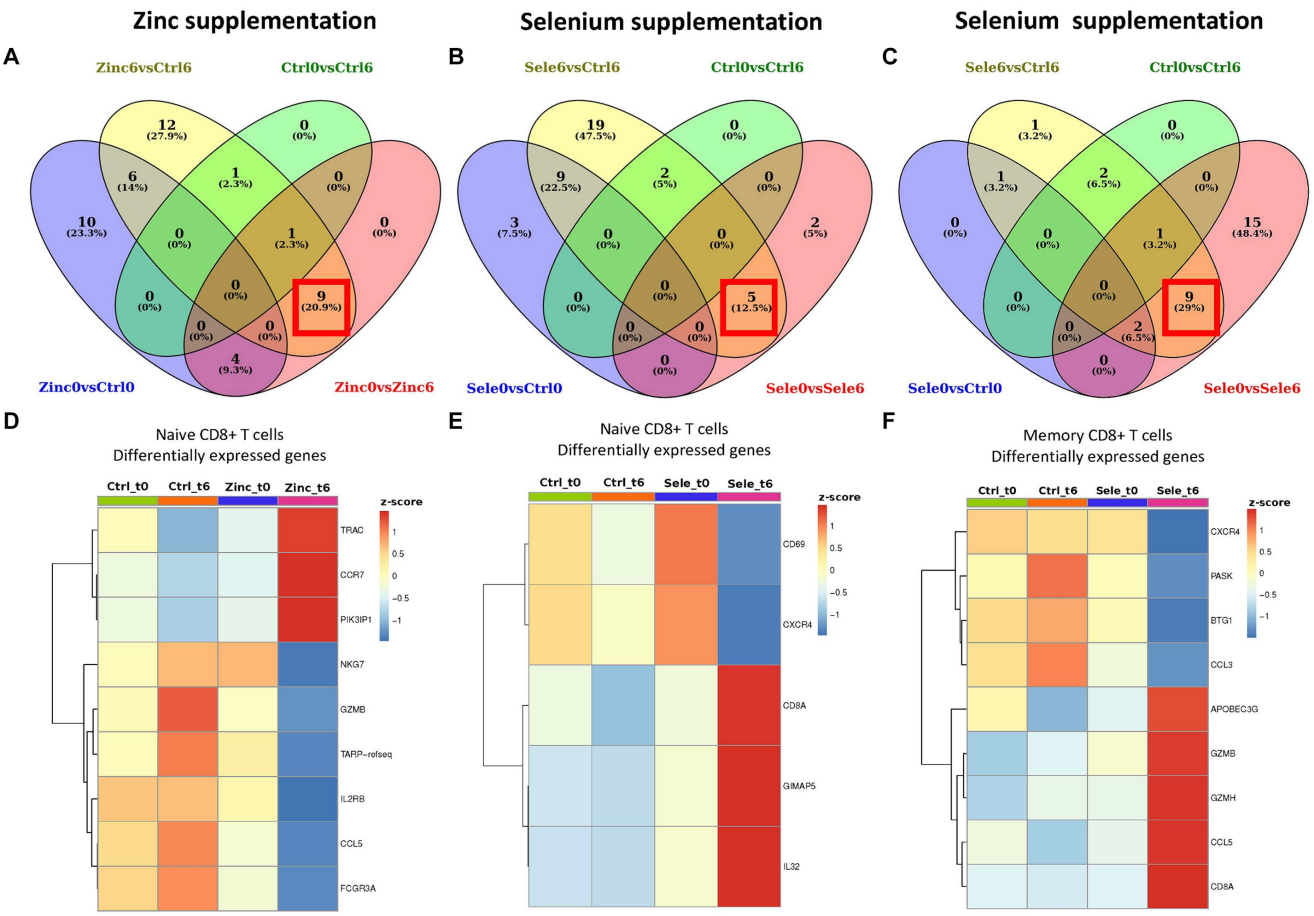
Differential gene expression analyses in longitudinal single cell RNA-sequencing data of zinc and selenium supplementation. Venn diagrams showing overlapping differentially expressed genes of different pairwise comparisons in naive CD8+ T cells after zinc supplementation **(A)** in naive CD8+ T cells after selenium supplementation **(B)** and in memory CD8+ T cells after selenium supplementation **(C)**. The red square shows the genes that we considered to be differentially expressed after 6 months supplementation with either zinc or selenium. The criteria used to considerer a gene differentially expressed is detailed in the methods section. Heatmaps showing the average expression of single cell RNA-sequencing differentially expressed genes induced after 6-month supplementation (t6) with zinc or selenium supplementation in naive T CD8+ cells **(D**,**E)** and memory T CD8+ cells **(F)**.

We explored the expression of selected immune-response-associated genes using a targeted single cell RNA-sequencing approach. This was evaluated in leukocytes, excluding neutrophils, from 1 individual that received 6 months of zinc supplementation, 1 individual that received 6 months of selenium and 1 individual that did not received any supplementation (control group), before (t0) and 6 months after receiving supplementation (t6; Figure 1). The individuals that were chosen for this experiment were the ones that experienced a significant increase of seric concentration of zinc and selenium after 6 months of supplementation. We did not find statistical differences on gene expression after supplementation in any of the immune populations: CD4+ T cells, monocytes, dendritic cells, gamma-delta T cells, mast cells, B cells, NK and NKT cells (data not shown). We considered a gene to be differentially expressed if its expression changed significantly in the supplemented group t0 vs. t6 and if it was differentially expressed compared to the control group (Figures 1A–C). We found that after 6 months of zinc supplementation, naïve CD8+ T cells significantly increased the expression of the genes: TRAC, CCR7, PIK3IP1 and decreased significantly the expression of the genes: NKG7, GZMB, TARP, IL2R, CCL5 and FCGR3A (adjusted *p* value <0.05 in all cases; Figure 1D; Supplementary Table 4). In the selenium supplemented group, we observed that after 6 months of supplementation, the naïve CD8+ T cells significantly decreased the expression of the genes: CD69 and CXCR4 and significantly increased the expression of the genes: CD8A, GIMAP5 and IL32 (*p* < 0.05 in all cases; Figure 1E; Supplementary Table 4). Also in the selenium supplemented group, we observed that the memory CD8+ T cells significantly decreased the expression of the genes: CXCR4, PASK, BTG1, and CCL3 and significantly increased the expression of the genes: APOBEC3G, GZMB, GZMMH, CCL5 and CD8A (*p* < 0.05 in all cases; Figure 1F; Supplementary Table 4).

## Discussion

In this study we observed that selenium supplementation has beneficial effects on body composition and on CD4+ T cell replenishment in PLWHIV on long-term ART without metabolic diseases. Additionally, zinc and selenium supplementation seem to modify the expression of genes associated with the function of naive and memory CD8+ T cells. All of these benefits were obtained without any adverse events such as nauseas, vomiting, diarrhea or dermatitis, with the dosage and presentation of the supplements used during the entire time of the intervention. To this date, we are not aware of any previous study that documented the effect of zinc and selenium supplementation on body composition, glucose and on lipid profile on clinically stable PLWHIV on long-term ART. Additionally, the effect of this intervention on the immune system at the transcriptome level has not been explored before.

First, we analyzed the effect of the supplementation on the body composition and bone mineral density (BMD). In our cohort, we did not observe significant changes in body composition or BMD after 6 months of zinc supplementation. In the case of the experimental group supplemented with selenium, we did not find any effect of supplementation on BMD, but we found a significant decrease in BMI and fat mass (almost 2 kg of fat) after 6 months of supplementation, this without a significant reduction of energy intake or an increase in physical activity during the supplementation period. One possible mechanism to explain this change on BMI and fat mass is the effect of selenium, particularly the selenoproteins glutathione peroxidase and selenoproteins P, on the regulation of enzymes of the insulin signaling cascade, the expression of lipogenic enzymes and in carbohydrate metabolism in the liver. Additionally, selenium promotes the formation of antioxidant selenoproteins, leading to reduced concentrations of reactive oxygen species, which are required in physiological amounts for appropriate insulin signaling (29–31). Unfortunately, we were unable to measure insulin or reactive oxygen species in our cohort. However, the reduction on fat mass may translate into substantial health benefits on PLWHIV, since high fat mass has been associated with metabolic diseases, cardiovascular risk and inflammation on treated PLWHIV (32). Follow-up studies with longer supplementation periods are necessary to determine the long-term benefits of selenium supplementation in the reduction of fat mass and the development of metabolic diseases.

After 6 months of zinc or/and selenium supplementation, we did not observe an effect on cholesterol, triglycerides, fasting glucose or blood pressure. We hypothesized that the intervention would be particularly beneficial to individuals with baseline seric zinc or selenium deficiency. After analyzing changes on the blood biochemical parameters only in the individuals with seric deficiency, we did not observe any effect of the supplementation. Further studies with increased number of individuals with zinc and selenium deficiency or with altered biochemical parameters are necessary in order to determine the effect of zinc and selenium supplementation on biochemical parameters. Additionally, there is a remarkable need for clinical research to study the effect of the dose and duration of the supplementation on the efficacy of nutritional interventions.

Interestingly, a previous study that analyzed the effect of zinc supplementation on PLWHIV on ART with sub-clinical inflammation, found that the response to supplementation was dependent on the absence of inflammation (33). In our cohort, we did not measure any marker of inflammation, but as HIV infection is characterized by chronic immune activation and inflammation, which is not reverted after ART (34), low grade inflammation may be an important factor that could affect supplementation effect on the biochemical parameters measured.

There are controversial reports about the effect of zinc and selenium supplementation on CD4+ T cell replenishment. In the case of zinc, some studies have reported no effect of zinc supplementation on CD4+ T cells levels (14). In contrast, Asdamongkol et al. and others, found increased CD4+ T cell counts after 6 months of zinc supplementation (16, 17), similar to the results obtained in our study with a tendency of increase CD4+ T cell counts after zinc supplementation. On the other hand, some authors have reported increased CD4+ T cell numbers after selenium supplementation, which is in line with our results, while others did not find any effect (20). The differences in the results obtained from the studies can be attributed to different doses and pharmacological presentation of the supplements, the duration of the intervention and the clinical/demographical characteristics of the cohort. It is essential to optimize the best dose and duration of supplementation to achieve benefits on CD4+ T cells recuperation using zinc and selenium interventions. In our study, the individuals that received selenium supplementation experienced an increase on CD4+ T cell frequency which represent an improvement on their health status and may be protective against apparition of opportunistic infections.

Persistent activation of the adaptative immune system, chronic inflammation and accelerated senescence have been associated with the development of non-AIDS comorbidities in PLWHIV on long-term ART (35). On clinically controlled long-term treated PLWHIV, reverting residual immune activation and inflammation to levels similar to those observed in non-HIV-infected individuals is one of the main objectives of clinical and basic research. As already demonstrated for vitamin D supplementation on treated HIV-infected individuals (36), we hypothesized that zinc and selenium could contribute to reducing immune activation and inflammation in our cohort, as previously reported elsewhere (21, 22). However, we did not find changes in the expression of activation markers CD38 and HLADR on CD4+ and CD8+ T cells after 6 months of zinc or/and selenium supplementation. It is necessary to continue looking for strategies to reduce and restore chronic inflammation and immune activation on treated PLWHIV.

Additionally, we hypothesized that at the cellular level, particularly in the transcriptome, the intervention would modify the expression of genes responsible for immune system responses, and that those changes could bring functional benefits that may not be reflected on the clinical measurements that we proposed as readouts. In fact, we found changes in the transcription of several genes in response to zinc and selenium supplementation, particularly in naïve CD8+ T cells, the cells of the adaptive immune system in charge of the response to novel pathogens, particularly to the antiviral and antitumoral immunity. In our targeted single cell RNA-sequencing experiment, we observed that after 6 months of zinc supplementation, naive CD8+ T cells increased the expression of genes associated with maturation (PIK3IP, TRAC), differentiation and migration to the lymph nodes (CCR7) where they develop immune responses to novel viral or tumoral antigens. These cells also decreased the expression of genes associated directly with their functional profile as degranulation (NKG7), cytotoxicity (GZMB), proliferation (IL2RB) and inflammation (CCL5). Also, after selenium supplementation, naïve CD8+ T cells upregulated the expression of genes associated with maturation (CD8a), activation (IL-32) and survival (GIMAP5). This could be interpretated as a beneficial effect of zinc or selenium supplementation, as naïve CD8+ T cells are expressing genes that prepare them to be successfully activated in case of encounter with novel viral or tumoral antigens, but, as expected for not experienced CD8+ T cells, they significantly reduced expression of genes directly associated with function in order to maintain the regulation of the immune response. Additionally, we found that selenium supplementation also has effect on memory CD8+ T cells, that are responsible for long-term maintenance of the immune response against viral or tumoral antigens. In this regard, our targeted single cell RNA-sequencing differential expression analysis showed that memory CD8+ T cells upregulated expression of genes associated with maturation (CD8A) and effector functions such as production of cytotoxic granules that directly kill virus infected or tumoral cells (GZMB, GZMH). Moreover, after selenium supplementation, memory CD8+ T cells significantly increased expression of genes associated with two of the major HIV-suppressive factors, the chemokine RANTES and the restriction factor APOBEC3G, which impair HIV infection of CD4+ T cells and causes hypermutation of the HIV genome during reverse transcription, respectively (37). These results warrant future research to study the effect of zinc and selenium supplementation at a cellular level, with a higher number of individuals per group and validation experiments to correlate gene expression with the presence of the functional protein.

It is well known that zinc and selenium have similar biological functions such as anti-inflammatory and antioxidant effect at a cellular level, therefore, we hypothesized the possibility of a synergistic interaction through their combined supplementation. The absence of effects after combining zinc and selenium supplementation on the clinical and immunological readouts of the present study might be associated with potential antagonism with other trace elements that compete for absorption sites and transporters. Moreover, altered homeostasis or impaired metabolism of other trace elements may impede correct zinc and selenium function (38). Therefore, it may be recommendable to assess the level of other trace elements, as cupper or iron, in order to improve micronutrient combination strategies to avoid possible antagonism between them that could decrease their clinical effect.

Our study has several limitations: (1) The statistical power of the study was limited by the reduced number of individuals in each intervention arm, which limited the study to a pilot clinical trial; (2) The control group did not receive placebo tablets and thus, the study was not blinded to this group participants; (3) Our data shows that a 6-month supplementation period may be insufficient to observe significant changes on the biochemical parameters evaluated in the present study. We cannot rule out the possibility that longer periods of supplementation could induce changes in the forementioned parameters; (4) Our observations may not be extrapolated to women, younger/older populations or ART naïve PLWHIV since all our cohort was composed by middle-aged men on long-term ART, which represents 90% of the PLWHIV that attend our institution; (5) We were not able to determine the seric zinc and selenium concentrations before supplementation. This was due to the requirement of specialized techniques for the measurement of micronutrients performed in a different institution, which resulted in the inclusion of participants with or without deficiency; (6) A longer follow-up period of the cohort is necessary in order to determine the long-term effect of zinc and selenium supplementation on the protection against opportunistic infections and the development of non-AIDS comorbidities; (7) For the single cell transcriptome experiments, we were able to include only one individual per group, and validation experiments were not carried out to confirm the results.

The present study brings some light into the understanding of the clinical, biochemical and immunological effect of zinc and selenium supplementation in clinically stable long-term treated PLWHIV without metabolic diseases or active coinfections. This population has been poorly studied and has not been considered as a target population for multiple interventions since they are unlikely to progress to AIDS, they have significantly increased their CD4+ T cell counts and immune responses. However, we consider that there are still multiple areas of opportunity to improve their health status and increase their quality of life. Several nutritional interventions have shown to be simple to implement, while also not interfering with ART, and to be economically feasible. We have shown the potential benefits of selenium supplementation on body composition, replenishment of CD4+ T cells and potentially beneficial transcriptional changes in CD8+ T cells that could improve negative effects associated with inflammation, metabolic and immune dysfunctions. Further research on other strategies to improve the quality of life and morbidity of PLWHIV on long-term ART are needed.

## Conclusion

Six months of selenium supplementation in middle-aged men PLWHIV on long-term ART has beneficial effects on body composition, specifically in reducing BMI and fat mass and with improved CD4+ T cell recuperation. Zinc or selenium or both micronutrients simultaneously supplemented during 6 months does not have an effect on total cholesterol, HDL or LDL levels, triglycerides or glucose. It is critical to determine the optimal duration of supplementation in order to improve the effect of nutritional interventions. Even on absence of changes on metabolic parameters, zinc or selenium supplementation is associated with the modification of the expression of genes responsible of the maturation of naive CD8+ T cells and with the antiviral capacity of memory CD8+ T cells, which could represent and improvement on their immune response. Zinc and selenium supplementation is a simple, economic and safe nutritional intervention that may complement the ART in order to improve the immune system function, health status and quality of life of PLWHIV on long-term ART clinically stable without metabolic diseases.

## Data Availability

The data from the single cell transcriptomics have been deposited in the repository GEO of the NCBI accession number GSE 253497 (https://www.ncbi.nlm.nih.gov/gds/?term=GSE253497). The rest of the data presented in the study can be found in the article and Supplementary material.

## References

[1] Shivakoti R Christian P Yang WT Gupte N Mwelase N Kanyama C. Prevalence and risk factors of micronutrient deficiencies pre- and post-antiretroviral therapy (ART) among a diverse multicountry cohort of HIV-infected adults. Clin Nutr. (2016) 35:183–9. doi: 10.1016/j.clnu.2015.02.002, PMID: 25703452 PMC4531105

[2] Shah KK Verma R Oleske JM Scolpino A Bogden JD. Essential trace elements and progression and management of HIV infection. Nutr Res. (2019) 71:21–9. doi: 10.1016/j.nutres.2019.08.001, PMID: 31668643

[3] Osuna-Padilla IA Briceño O Aguilar-Vargas A Rodríguez-Moguel NC Villazon-De la Rosa A Pinto-Cardoso S. Zinc and selenium indicators and their relation to immunologic and metabolic parameters in male patients with human immunodeficiency virus. Nutrition. (2020) 70:110585. doi: 10.1016/j.nut.2019.110585, PMID: 31698296

[4] Read SA Obeid S Ahlenstiel C Ahlenstiel G. The role of zinc in antiviral immunity. Adv Nutr. (2019) 10:696–710. doi: 10.1093/advances/nmz013, PMID: 31305906 PMC6628855

[5] Campa A Shor-Posner G Indacochea F Zhang G Lai H Asthana D. Mortality risk in selenium-deficient HIV-positive children. J Acquir Immune Defic Syndr Hum Retrovirol. (1999) 20:508–13. doi: 10.1097/00042560-199904150-00015, PMID: 10225235

[6] Steinbrenner H Duntas LH Rayman MP. The role of selenium in type-2 diabetes mellitus and its metabolic comorbidities. Redox Biol. (2022) 50:102236. doi: 10.1016/j.redox.2022.102236, PMID: 35144052 PMC8844812

[7] Sapota A Daragó A Skrzypińska-Gawrysiak M Nasiadek M Klimczak M Kilanowicz A. The bioavailability of different zinc compounds used as human dietary supplements in rat prostate: a comparative study. Biometals. (2014) 27:495–505. doi: 10.1007/s10534-014-9724-9, PMID: 24619814

[8] Burk RF Norsworthy BK Hill KE Motley AK Byrne DW. Effects of chemical form of selenium on plasma biomarkers in a high-dose human supplementation trial. Cancer Epidemiol Biomarkers Prev. (2006) 15:804–10. doi: 10.1158/1055-9965.EPI-05-0950, PMID: 16614127

[9] Kamwesiga J Mutabazi V Kayumba J Tayari JCK Uwimbabazi JC Batanage G. Effect of selenium supplementation on CD4+ T-cell recovery, viral suppression and morbidity of HIV-infected patients in Rwanda: a randomized controlled trial. AIDS. (2015) 29:1045–52. doi: 10.1097/QAD.0000000000000673, PMID: 25870994 PMC4444428

[10] Green JA Lewin SR Wightman F Lee M Ravindran TS Paton NI. A randomised controlled trial of oral zinc on the immune response to tuberculosis in HIV-infected patients. Int J Tuberc Lung Dis. (2005) 9:1378–84. PMID: 16466061

[11] Cárcamo C Hooton T Weiss NS Gilman R Wener MH Chavez V. Randomized controlled trial of zinc supplementation for persistent diarrhea in adults with HIV-1 infection. J Acquir Immune Defic Syndr. (2006) 43:197–201. doi: 10.1097/01.qai.0000242446.44285.b5, PMID: 16940855

[12] Zeng L Zhang L. Efficacy and safety of zinc supplementation for adults, children and pregnant women with HIV infection: systematic review. Trop Med Int Health. (2011) 16:1474–82. doi: 10.1111/j.1365-3156.2011.02871.x, PMID: 21895892

[13] Baum MK Campa A Lai S Sales Martinez S Tsalaile L Burns P. Effect of micronutrient supplementation on disease progression in asymptomatic, antiretroviral-naive, HIV-infected adults in Botswana: a randomized clinical trial. JAMA. (2013) 310:2154–63. doi: 10.1001/jama.2013.280923, PMID: 24281460 PMC4347896

[14] Hadadi A Ostovar A Edalat Noor B Rasoolinejad M Haji Abdolbaghi M Yousefi S. The effect of selenium and zinc on CD4(+) count and opportunistic infections in HIV/AIDS patients: a randomized double blind trial. Acta Clin Belg. (2020) 75:170–6. doi: 10.1080/17843286.2019.1590023, PMID: 30888253

[15] Mocchegiani E Veccia S Ancarani F Scalise G Fabris N. Benefit of oral zinc supplementation as an adjunct to zidovudine (AZT) therapy against opportunistic infections in AIDS. Int J Immunopharmacol. (1995) 17:719–27. doi: 10.1016/0192-0561(95)00060-f, PMID: 8582783

[16] Asdamongkol N Phanachet P Sungkanuparph S. Low plasma zinc levels and immunological responses to zinc supplementation in HIV-infected patients with immunological discordance after antiretroviral therapy. Jpn J Infect Dis. (2013) 66:469–74. doi: 10.7883/yoken.66.469, PMID: 24270132

[17] Silva M Montes CG Canals A Mackenna MJ Wolff M. Role and effects of zinc supplementation in HIV-infected patients with immunovirological discordance: a randomized, double blind, case control study. PLoS One. (2021) 16:e0244823. doi: 10.1371/journal.pone.0244823, PMID: 33481813 PMC7822263

[18] Bao B Prasad AS Beck FWJ Fitzgerald JT Snell D Bao GW. Zinc decreases C-reactive protein, lipid peroxidation, and inflammatory cytokines in elderly subjects: a potential implication of zinc as an atheroprotective agent. Am J Clin Nutr. (2010) 91:1634–41. doi: 10.3945/ajcn.2009.28836, PMID: 20427734 PMC2869512

[19] Hurwitz BE Klaus JR Llabre MM Gonzalez A Lawrence PJ Maher KJ. Suppression of human immunodeficiency virus type 1 viral load with selenium supplementation: a randomized controlled trial. Arch Intern Med. (2007) 167:148–54. doi: 10.1001/archinte.167.2.148, PMID: 17242315

[20] Muzembo BA Ngatu NR Januka K Huang HL Nattadech C Suzuki T. Selenium supplementation in HIV-infected individuals: a systematic review of randomized controlled trials. Clin Nutr ESPEN. (2019) 34:1–7. doi: 10.1016/j.clnesp.2019.09.005, PMID: 31677697

[21] Hariharan S Dharmaraj S. Selenium and selenoproteins: it’s role in regulation of inflammation. Inflammopharmacology. (2020) 28:667–95. doi: 10.1007/s10787-020-00690-x 32144521 PMC7222958

[22] Jafari A Ghobadi S. Zinc supplementation and inflammatory cytokines. Br J Nutr. (2022) 127:478. doi: 10.1017/S0007114521001069, PMID: 33762033

[23] Zicari S Sessa L Cotugno N Ruggiero A Morrocchi E Concato C. Immune activation, inflammation, and non-AIDS co-morbidities in HIV-infected patients under long-term ART. Viruses. (2019) 11:200. doi: 10.3390/v11030200, PMID: 30818749 PMC6466530

[24] Herrera KR. Guía para la detección del Virus de la Inmunodeficiencia Humana (VIH). México: Censida/Secretaría de Salud (2018).

[25] Berger MM Shenkin A Dizdar OS Amrein K Augsburger M Biesalski HK. ESPEN practical short micronutrient guideline. Clin Nutr. (2024) 43:825–57. doi: 10.1016/j.clnu.2024.01.030, PMID: 38350290

[26] Alberti KGMM Eckel RH Grundy SM Zimmet PZ Cleeman JI Donato KA. Harmonizing the metabolic syndrome: a joint interim statement of the international diabetes federation task force on epidemiology and prevention; National Heart, Lung, and Blood Institute; American Heart Association; World Heart Federation; International Atherosclerosis Society; and International Association for the Study of obesity. Circulation. (2009) 120:1640–5. doi: 10.1161/CIRCULATIONAHA.109.192644, PMID: 19805654

[27] Dobin A Davis CA Schlesinger F Drenkow J Zaleski C Jha S. STAR: ultrafast universal RNA-seq aligner. Bioinformatics. (2013) 29:15–21. doi: 10.1093/bioinformatics/bts635, PMID: 23104886 PMC3530905

[28] Hao Y Hao S Andersen-Nissen E Mauck WM Zheng S Butler A. Integrated analysis of multimodal single-cell data. Cell. (2021) 184:3573–3587.e29. doi: 10.1016/j.cell.2021.04.048, PMID: 34062119 PMC8238499

[29] Steinbrenner H. Interference of selenium and selenoproteins with the insulin-regulated carbohydrate and lipid metabolism. Free Radic Biol Med. (2013) 65:1538–47. doi: 10.1016/j.freeradbiomed.2013.07.016, PMID: 23872396

[30] Mao J Teng W. The relationship between selenoprotein P and glucose metabolism in experimental studies. Nutrients. (2013) 5:1937–48. doi: 10.3390/nu5061937, PMID: 23760059 PMC3725484

[31] Iizuka Y Ueda Y Yagi Y Sakurai E. Significant improvement of insulin resistance of GK rats by treatment with sodium selenate. Biol Trace Elem Res. (2010) 138:265–71. doi: 10.1007/s12011-010-8622-4, PMID: 20177813

[32] Bonjoch A de Cabo F Puig J Perez-Alvarez N Echeverria P Clotet B. Ultrasound-based assessment of preperitoneal fat as a surrogate marker of cardiovascular risk: comparative study between people living with HIV and controls. AIDS Res Hum Retrovir. (2022) 38:222–7. doi: 10.1089/aid.2021.0141, PMID: 34969253

[33] Mburu ASW Thurnham DI Mwaniki DL Muniu EM Alumasa FM. The influence of inflammation on plasma zinc concentration in apparently healthy, HIV+ Kenyan adults and zinc responses after a multi-micronutrient supplement. Eur J Clin Nutr. (2010) 64:510–7. doi: 10.1038/ejcn.2010.33, PMID: 20216563

[34] Hileman CO Funderburg NT. Inflammation, immune activation, and antiretroviral therapy in HIV. Curr HIV/AIDS Rep. (2017) 14:93–100. doi: 10.1007/s11904-017-0356-x, PMID: 28434169 PMC5514315

[35] Duffau P Ozanne A Bonnet F Lazaro E Cazanave C Blanco P. Multimorbidity, age-related comorbidities and mortality: association of activation, senescence and inflammation markers in HIV adults. AIDS. (2018) 32:1651–60. doi: 10.1097/QAD.0000000000001875, PMID: 29762168

[36] Fabre-Mersseman V Tubiana R Papagno L Bayard C Briceno O Fastenackels S. Vitamin D supplementation is associated with reduced immune activation levels in HIV-1-infected patients on suppressive antiretroviral therapy. AIDS. (2014) 28:2677–82. doi: 10.1097/QAD.0000000000000472, PMID: 25493593

[37] Perdomo-Celis F Taborda NA Rugeles MT. CD8+ T-cell response to HIV infection in the era of antiretroviral therapy. Front Immunol. (2019) 10:1896. doi: 10.3389/fimmu.2019.01896, PMID: 31447862 PMC6697065

[38] Bjørklund G Aaseth J Skalny AV Suliburska J Skalnaya MG Nikonorov AA. Interactions of iron with manganese, zinc, chromium, and selenium as related to prophylaxis and treatment of iron deficiency. J Trace Elem Med Biol. (2017) 41:41–53. doi: 10.1016/j.jtemb.2017.02.005, PMID: 28347462

