# Supplementary material for: Zinc and selenium supplementation on treated HIV-infected individuals induces changes in body composition and on the expression of genes responsible of naïve CD8+ T cells function
Source: Front Nutr. 2024 Sep 16;11:1417975. doi: 10.3389/fnut.2024.1417975 (PMC11439712; doi:10.3389/fnut.2024.1417975)
Supplement: Supplementary file 1 [file Table_1.DOCX]

**Supplementary Table 1. Body composition, Biochemical and Clinical characteristics of the cohort before supplementation.**

| **A.- Body composition and bone mineral density measurements** | | | | | |
| --- | --- | --- | --- | --- | --- |
|  | **^G1 assigned to zinc supplementation^**  **^(n=18)^** | **^G2 assigned to selenium supplementation (n=13)^** | **^G3 assigned to zinc+selenium supplementation^**  **^(n=13)^** | **^G4 assigned to control^**  **^No supplementation (n=16)^** | **P value** |
| **Weight (kg)** | 66.3 ± 7.9 | 70.9 ± 10.9 | 68.6 ± 7.7 | 71.8 ± 12.0 | 0.38^a^ |
| **BMI (kg/m^2^)** | 23.0 ± 2.6 | 24.6 ± 4.1 | 23.7 ± 2.8 | 24.4 ± 3.4 | 0.52^a^ |
| **Fat mass (%)**  **Fat mass (kg)** | 23.9 ± 3.8  16.1 ± 3.8 | 28.6 ± 4.5  20.6 ± 6.3 | **23.0 ± 5.3**  **16.0 ± 5.0** | **27.1 ± 5.8**  **19.7 ± 6.1** | **0.01^a⁺^**  **0.03^aΔ^** |
| **Trunk fat mass (kg)** | 9.1 ± 2.8 | 11.6 ± 4.0 | 8.9 ± 3.3 | 11.3 ± 4.0 | 0.08^a^ |
| **Muscle mass (kg)** | 48.2 ± 5.7 | 47.5 ± 5.4 | 50.3 ± 5.4 | 49.5 ± 7.7 | 0.65^a^ |
| **Femoral BMD (g/cm^2^)** | 0.93 ± 0.14 | 1.02 ± 0.15 | 0.96 ± 0.09 | 0.99 ± 0.07 | 0.40 ^a^ |
| **Lumbar BMD (g/cm^2^)** | 1.04 ± 0.14 | 1.17 ± 0.13 | **1.10 ± 0.10** | **1.12 ± 0.11** | **0.03^a^‡** |
| **B.- Biochemical measurements** | | | | | |
| **Total cholesterol (mg/dL)** | 168.3 ± 33.8 | 185 ± 39.7 | 182.5 ± 28.3 | 175.1 ± 42.8 | 0.58^a^ |
| **Hypercholesterolemia**  **(>200 mg/dL)** | 2 (11%) | 4 (31%) | 4 (31%) | 5 (31%) | 0.44 |
| **HDL cholesterol (mg/dL)** | 42.8 ± 8.7 | 44.0 ± 7.1 | 43.5 ± 11.7 | 43.5 ± 9.1 | 0.98^a^ |
| **Low HDL**  **(<40 mg/dL)** | 6 (33%) | 4 (31%) | 6 (46%) | 6 (37%) | 0.85 |
| **LDL cholesterol (mg/dL)** | 100.3 ± 27.8 | 115.1 ± 32.4 | 113 ± 22.6 | 103.9 ± 32.0 | 0.44^a^ |
| **High LDL**  **(>110 mg/dL)** | 8 (44%) | 7 (54%) | 7 (54%) | 8 (50%) | 0.94 |
| **Triglycerides**  **(mg/dL)** | 139 (97-269) | 138 (81-164) | 164 (105-228) | 125 (100-266) | 0.84^b^ |
| **Hypertriglicerydemia**  **(>150 mg/dL)** | 7 (39%) | 5 (38%) | 7 (54%) | 6 (38%) | 0.79 |
| **Glucose**  **(mg/dL)** | 90 ± 13.3 | 87.9 ± 12.7 | 89.7 ± 5.9 | 91.7 ± 9.5 | 0.12^a^ |
| **High glucose**  **(>100 mg/dL)** | 3 (16%) | 1 (8%) | 1 (8%) | 2 (12.5%) | 0.83 |
| **C.- Clinical measurements** | | | | | |
| **Systolic Blood pressure (mmHg)**  **Diastolic Blood pressure (mmHg)** | 113 ± 10  66 ± 10 | 112 ± 10  68 ± 7 | 116 ± 10  70 ± 8 | 115 ± 10  70 ± 6 | 0.99^a^  0.49^a^ |

a: one way ANOVA, b: Kruskal-Wallis test, c: *X*^2^ test; ⁺ p<0.05 between G2 vs G3; Δ no differences in Bonferroni post-hoc test; ‡ p<0.05 between G1 vs G2.

BMI: Body mass index, BMD: Bone Mineral Density, HDL: High Density lipoprotein, LDL: Low density lipoprotein.

**Supplementary Table 2. Changes in biochemical parameters after zinc supplementation in individuals with baseline seric zinc deficiency**

|  | **G1= Zinc**  **supplemented group**  **(n=4)** | | | **G3= Zinc + Selenium supplemented group**  **(n=1)** | | | **G4= No supplemented**  **Control group**  **(n=5)** | | |
| --- | --- | --- | --- | --- | --- | --- | --- | --- | --- |
|  | Baseline | Six-Months | P | Baseline | Six-Months | P | Baseline | Six-Months | P |
| **Total cholesterol (mg/dL)** | 144.3 ± 37.8 | 149.6 ± 43.1 | 0.69 | 179 | 171 | 0.90 | 166.2 ± 127.7 | 162.7 ± 145.2 | 0.75 |
| **HDL- Cholesterol (mg/dL)** | 43.5 ± 10.3 | 42.0 ± 10.7 | 0.50 | 45 | 44.8 | ND | 42.5 ± 2.3 | 41.8 ± 3.2 | 0.59 |
| **LDL- cholesterol**  **(mg/dl)** | 87.5 ± 28.7 | 93.8 ± 24.8 | 0.53 | 123.8 | 116.1 | ND | 104.6 ± 23.6 | 97.2 ± 8.8 | 0.49 |
| **Triglycerides**  **(mg/dL)** | 132  (91-150) | 120  (75.5-188) | 0.71 | 237.5 | 74.5 | 0.31 | 107.5  (105.4-117) | 112.7  (100-143) | 0.89 |
| **Glucose**  **(mg/dL)** | 102.9  (90.8-118.6) | 97.7  (82.7-106.5) | 0.14 | 89 | 85.4 | 0.31 | 86.2  (86-88) | 88  (84-90) | 0.49 |
| **CD4+ (cells/mm3)** | 419  (282-730) | 536  (333-989) | 0.06 | 853 | 1029 | 0.31 | 485  (470-506) | 598  (456-732) | 0.22 |
| **CD4 (%)** | 30.2 ± 12.6 | 30.7 ± 12.0 | 0.49 | 41 | 33 | ND | 31 ± 6.1 | 32.4 ± 8.1 | 0.41 |

Wilcoxon test was performed for comparison before and after supplementation. ND: Non determined; HDL: High Density lipoprotein, LDL: Low density lipoprotein.

**Supplementary Table 3. Changes in biochemical parameters after selenium supplementation in individuals with baseline seric selenium deficiency.**

|  | **G2= Selenium**  **supplemented group**  **(n=4)** | | | **G3= Zinc + Selenium supplemented group**  **(n=7)** | | | **G4= No supplemented**  **Control group**  **(n=8)** | | |
| --- | --- | --- | --- | --- | --- | --- | --- | --- | --- |
|  | Baseline | Six-Months | P | Baseline | Six-Months | P | Baseline | Six-Months | P |
| **Total cholesterol (mg/dL)** | 181.2 ± 22.9 | 178.5 ± 15.8 | 0.74 | 179.3 ± 36.2 | 200.9 ±25.4 | 0.08 | 177.4 ± 44.5 | 186.6 ± 47.6 | 0.28 |
| **HDL- Cholesterol (mg/dL)** | 46 ± 7.0 | 49 ± 12.7 | 0.39 | 35.2 ± 6.9 | 40.4 ± 6.7 | 0.06 | 44.7 ± 10.0 | 42.6 ± 5.4 | 0.60 |
| **LDL- cholesterol**  **(mg/dl)** | 110.7 ± 28.0 | 104.7 ± 21.6 | 0.35 | 111.2 ± 25.7 | 123.4 ± 14.9 | 0.24 | 109.1 ± 37.7 | 118.1 ± 38.6 | 0.21 |
| **Triglycerides**  **(mg/dL)** | 138  (105-149) | 84  (77-134) | 0.46 | 198  (121-249) | 206  (128-281) | 0.73 | 120  (105-202) | 144  (124-157) | 0.40 |
| **Glucose**  **(mg/dL)** | 84  (81-87) | 87  (83-89) | 0.85 | 88  (86-6) | 90  (89-93) | 0.15 | 92  (85-98) | 90  (86-98) | 0.88 |
| **CD4+ (cells/mm3)** | 433  (312-567) | 453  (305-581 | 0.71 | 404  (307-468) | 379  (318-491) | 0.86 | 650  (456-891) | 635  (416-843) | 0.44 |
| **CD4 (%)** | 26.2 ± 14.0 | 31 ± 16.8 | 0.11 | 25.5 ± 8.5 | 26.2 ± 5.9 | 0.57 | 28.6 ± 11.0 | 30.3 ± 11.3 | 0.09 |

Wilcoxon test was performed for comparison before and after supplementation. HDL: High Density lipoprotein, LDL: Low density lipoprotein.

**Supplementary table 4. Function of the genes differentially expressed after zinc or selenium supplementation.**

| **Zinc supplementation – Genes differentially expressed on Naïve CD8+ T cells** | | | |
| --- | --- | --- | --- |
| **Gene** | **Function** | **Adjusted p-value** |  |
| TRAC | T cell receptor alpha constant. | 2.7846E-06 |  |
| CCR7 | Naïve and memory CD8+ T express high levels of CCR7 hence they can migrate to the T cell zone of the lymph nodes and spleen in order to be activated in the T cell zone by the antigen presenting cells and differentiate into effector cells. | 7.499E-06 |  |
| PIK3IP1 | Induce the maturation and capacity of CD8+ cytotoxic T lymphocytes (CTLs) to kill tumoral and virus infected cells. | 0.00019428 |  |
| NKG7 | NKG7 function in natural killer and CD8^+^ T cells and was linked with their ability to regulate the translocation of CD107a to the cell surface to induce degranulation and kill cellular targets. | 2.9636E-06 |  |
| GZMB | This gene encodes a member of the granzyme subfamily of proteins, part of the peptidase S1 family of serine proteases. The encoded preproprotein is secreted by natural killer (NK) cells and cytotoxic T lymphocytes (CTLs) and proteolytically processed to generate the active protease, which induces target cell apoptosis. | 2.5631E-06 |  |
| TARP-refseq | T Cell Receptor Gamma Chain Variable. | 0.0394642 |  |
| IL2RB | The interleukin 2 receptor subunit Beta is involved in T cell-mediated immune responses and proliferation. | 0.00506813 |  |
| CCL5 | Beta-Chemokine RANTES. Secreted protein involved in immunoregulatory and inflammatory processes. Functions as a chemoattractant for blood monocytes, memory T helper cells and eosinophils. It functions as one of the natural ligands for the chemokine receptor chemokine (C-C motif) receptor 5 (CCR5), and it suppresses in vitro replication of the R5 strains of HIV-1, which use CCR5 as a coreceptor. | 0.01922579 |  |
| FCGR3A | Immunoglobulin G Fc Receptor III is involved in the removal of antigen-antibody complexes from the circulation, as well as other responses, including antibody dependent cellular mediated cytotoxicity and antibody dependent enhancement of virus infections. | 0.01518748 |  |
| **Selenium supplementation- Genes differentially expressed on Naïve CD8+ T cells** | | | |
| CD69 | Expression of the encoded protein is induced upon activation of T lymphocytes, and may play a role in proliferation. | 0.00095683 |  |
| CXCR4 | This gene encodes a CXC chemokine receptor specific for stromal cell-derived factor-1. It acts with the CD4 protein to support HIV entry into cells. | 1.6708E-10 |  |
| CD8A | The CD8 antigen is a cell surface glycoprotein found on most cytotoxic T lymphocytes that mediates efficient cell-cell interactions within the immune system. The CD8 antigen acts as a coreceptor with the T-cell receptor on the T lymphocyte to recognize antigens displayed by an antigen presenting cell in the context of class I MHC molecules. | 0.04384472 |  |
| GIMAP5 | This gene encodes a protein belonging to the GTP-binding superfamily and to the immuno-associated nucleotide (IAN) subfamily of nucleotide-binding proteins. This gene encodes an antiapoptotic protein that functions in T-cell survival. | 0.02954785 |  |
| IL32 | Expression of this protein is increased after the activation of T-cells by mitogens. This protein induces the production of TNF-a from macrophage cells. | 3.7185E-08 |  |
| **Selenium supplementation- Genes differentially expressed on Memory CD8+ T cells** | | | |
| CXCR4 | This gene encodes a CXC chemokine receptor specific for stromal cell-derived factor-1. It acts with the CD4 protein to support HIV entry into cells. | 1.0623E-21 |  |
| PASK | This gene encodes a member of the serine/threonine kinase family that contains two PAS domains. Expression of this gene is regulated by glucose, and the encoded protein plays a role in the regulation of insulin gene expression. Downregulation of this gene may play a role in type 2 diabetes. | 2.8957E-06 |  |
| BTG1 | This gene is a member of an anti-proliferative gene family that regulates cell growth and differentiation. Expression of this gene is highest in the G0/G1 phases of the cell cycle and downregulated when cells progressed through G1. The encoded protein interacts with several nuclear receptors, and functions as a coactivator of cell differentiation. | 4.72E-06 |  |
| CCL3 | The encoded protein, also known as macrophage inflammatory protein 1 alpha, plays a role in inflammatory responses through binding to the receptors CCR1, CCR4 and CCR5. Polymorphisms at this locus may be associated with both resistance and susceptibility to infection by human immunodeficiency virus type 1. | 3.9282E-06 |  |
| APOBEC3G | This gene is a member of the cytidine deaminase gene family. Members of the cluster encode proteins that are structurally and functionally related to the C to U RNA-editing cytidine deaminase APOBEC1. The protein encoded by this gene catalyzes site-specific deamination of both RNA and single-stranded DNA. The encoded protein has been found to be a specific inhibitor of human immunodeficiency virus-1 (HIV-1) infectivity. | 3.9148E-05 |  |
| GZMB | This gene encodes a member of the granzyme subfamily of proteins, part of the peptidase S1 family of serine proteases. The encoded preproprotein is secreted by natural killer (NK) cells and cytotoxic T lymphocytes (CTLs) and proteolytically processed to generate the active protease, which induces target cell apoptosis. | 0.0001558 |  |
| GZMH | Cytotoxic T-Lymphocyte-Associated Serine Esterase. This protein is reported to be constitutively expressed in the NK (natural killer) and induced expression on CTLs CD8+ T cells inducing target cell death and by directly cleaving substrates in virus-infected cells. | 9.1424E-07 |  |
| CCL5 | Beta-Chemokine RANTES. Secreted proteins involved in immunoregulatory and inflammatory processes. It functions as a chemoattractant for blood monocytes, memory T helper cells and eosinophils.  Is one of the major HIV-suppressive factors produced by CD8+ cells. It functions as one of the natural ligands for the chemokine receptor chemokine (C-C motif) receptor 5 (CCR5), and it suppresses in vitro replication of the R5 strains of HIV-1, which use CCR5 as a coreceptor. | 2.8155E-09 |  |
| CD8A | The CD8 antigen is a cell surface glycoprotein found on most cytotoxic T lymphocytes that mediates efficient cell-cell interactions within the immune system. The CD8 antigen acts as a coreceptor with the T-cell receptor on the T lymphocyte to recognize antigens displayed by an antigen presenting cell in the context of class I MHC molecules. | 6.8391E-11 |  |

The function of all the genes were obtained from GenBank https://www.ncbi.nlm.nih.gov/genbank/
